# Two successive pregnancies in a patient during 14 years of hemodialysis: a case report

**DOI:** 10.1186/s13256-016-0836-4

**Published:** 2016-03-08

**Authors:** Ayse Seker

**Affiliations:** Department of Nephrology, Kars State Hospital, Kars, Turkey

**Keywords:** End stage renal disease, Hemodialysis, Two successful pregnancies, Vaginal delivery, Case report

## Abstract

**Background:**

The incidence of pregnancy in women with end-stage renal disease seems to be increasing. Improvements in dialysis, obstetrical care, and antenatal fetal monitoring over the past two decades appear to have increased fertility rates and successful pregnancies in dialysis-dependent women. A pregnancy with a successful outcome despite the long dialysis period, as in the patient described here, is very rarely reported in the literature.

**Case presentation:**

I report a case of a 34-year-old white Kurdish woman who had had two uncomplicated pregnancies while on hemodialysis, with delivery of healthy babies. The first pregnancy occurred in the eighth year of her hemodialysis and ended in the 32nd week of gestation with healthy vaginal delivery of a 1900-g baby. The second pregnancy occurred in the 14th year of her hemodialysis and ended in the 30th week of gestation, with vaginal delivery of a 1400-g baby. The second baby, who had respiratory problems, was discharged after 45 days of intensive care.

**Conclusions:**

Although pregnancy during the hemodialysis is risky, the outcomes of the pregnancies could be improved by an intensive hemodialysis regimen, appropriate anemia management, strict blood pressure follow-up, and correct evaluation of the dry weight.

## Background

Women of childbearing age seldom become pregnant while on chronic dialysis, owing to disturbances in the hypothalamic-pituitary-ovarian axis and other associated psychological factors [1, 2]. The incidence of pregnancy, defined by a gestational age of 3 months or more, was reported in one study to be only 0.3 per 100 patient-years over a period of 20 years, even in women of reproductive age; the successful live birth rate was less than 60 % [3]. The risk of intrauterine growth retardation, polyhydramnios, and premature rupture of membranes in this clinical setting is increased. Most living babies are born prematurely. Although advances in dialysis technology, obstetrical monitoring, and neonatal intensive care over the last 20 years have dramatically improved fetal survival rates to roughly 80 %, fetal mortality in pregnant women on dialysis is still much higher than in the general population [4]. Our patient, despite long duration of dialysis (first pregnancy in 8th year and second in 14th year of dialysis) gave birth to two healthy babies, which is rarely reported in the literature.

## Case presentation

### First pregnancy

A 34-year-old white Kurdish woman, gravida 4, para 3, has been on regular hemodialysis in our center for 14 years due to hypertensive nephrosclerosis. She had cerebrovascular disease at the age of 20 years and at that time was diagnosed with hypertension and end-stage renal disease. She was started on hemodialysis 4 h/day, 3 days/week. She was admitted to the author’s clinic with suspected pregnancy in the 8th year of dialysis. A pregnancy at 14 weeks of gestation was been detected on her pelvic ultrasound. She was receiving cilazapril 5 mg/day for hypertension, which was discontinued after diagnosis of pregnancy. She underwent hemodialysis 5 h/day, 6 days/week until the end of her pregnancy. Her dialysis access was via an arteriovenous fistula, and conventional hemodialysis was performed. She did not need antihypertensive medication during this pregnancy. She was given recombinant human erythropoietin, intravenous iron, calcium, and folic acid. In the 32nd week of gestation, she was admitted to the hospital with early membrane rupture. Intramuscular betamethasone was administered twice with an interval of 24 h to induce fetal lung maturation. The patient subsequently delivered a healthy, 1900-g girl. In the postpartum follow-up, neither the mother nor the baby developed a complication.

### Second pregnancy

In the 14th year of the patient’s dialysis treatment, she presented with amenorrhea of 3 months’ duration. Her β-human chorionic gonadotropin blood test result was positive, and a pelvic ultrasound confirmed a single 12-week gestational age fetus. The patient was taking valsartan 160 mg/day and amlodipine 10 mg/day for hypertension. Valsartan was discontinued after her pregnancy diagnosis. Her dialysis regimen was intensified to a regimen consisting of 30 h/week (5-h treatment sessions on 6 days/week). During dialysis, 1.5 m^2^ polysulfone and a high-flux dialyzer were used and blood flow rate was kept at a level of 280 ml/minute on average. The patient was started on a 2000 kcal/day, 1.5 g/kg/day protein-rich diet. She was anticoagulated with unfractionated heparin. Her blood pressure (BP) was well controlled after intensive hemodialysis, and amlodipine was discontinued. Without any antihypertensive medication, her systolic BP remained at 110–140 mmHg and her diastolic BP was consistently 70–90 mmHg. Anemia was managed with subcutaneous recombinant human erythropoietin three times per week and intravenous iron without transfusion. The median erythropoietin dose was 12,000 international units (IU)/week (range 6,000–18,000 IU/week). Folic acid 1 mg/day and calcium 1500 mg/day were added to her medications. Dialysate bicarbonate content of 30 mEq/L was set to prevent pregnancy-induced respiratory alkalosis. The dialysate contained 1 g/L of glucose, 1.25 mEq/L of calcium, and 3 mEq/L of potassium. The estimated dry weight of the patient was increased according to her weight gain. Her dry weight was 54 kg in the beginning of pregnancy, and her weight gain was 9 kg during the pregnancy. Ultrasonography revealed polyhydramnios at 28 weeks.

In the 30th week of pregnancy, the patient’s contractions began and she had a preterm vaginal delivery of a boy weighing 1400 g. The baby, whose Apgar scores were 5 at the 1st minute and 8 at the 5th minute was taken to the intensive care unit because of his respiratory problem. Forty-five days later, the baby was discharged healthy.

## Discussion

According to data obtained from the European Dialysis and Transplant Association (EDTA) and national registries, the conception rate for women receiving dialysis is between 0.3 and 0.75/year/patient [3, 5]. The possibility of conception and successful conclusion of pregnancy are much higher in women who have residual renal function, and the prognosis for pregnancies is better if conception has occurred before starting dialysis. Giatras *et al*. [6] demonstrated that 47 % of pregnancies among women in their population were successful during the first 2 years of dialysis, whereas only 6 of the 120 pregnancies of women with 10 years of treatment had successful outcomes. In the literature, there are reports of pregnancies of women with 10 years of dialysis, but positive outcomes of these pregnancies are extremely rare [5, 7, 8]. It has been shown that the prognosis for successful conclusion of pregnancy is better for patients who begin dialysis after the onset of pregnancy as compared with patients who are already on dialysis (72.6 % vs 37.5 %, respectively) [4]. As for my patient, she conceived twice, the first time 8 years and the second time 14 years after she had started hemodialysis. The first baby was discharged without a need for intensive care, and the second was discharged healthy after 45 days of intensive care. A pregnancy with a successful outcome despite the long dialysis period as in my patient is very rarely reported in the literature. Several potential determinants of the pregnancy outcomes in such patients are residual renal function, parity, age, and maternal diseases such as hypertension. Although my patient was hypertensive during 14 years of dialysis, she was normotensive in her pregnancies. The reason for this may be 5 h/day, 6 days/week intensive hemodialysis and correct evaluation of dry weight.

The literature contains two reports of cases of three successive pregnancies while the patients were on hemodialysis [9, 10]. In a case report by Malik *et al.*, only the first pregnancy was full-term and successful. The second pregnancy ended in premature delivery. The third pregnancy ended with antepartum hemorrhage due to placenta previa and rupture of the uterus [9]. The babies died after birth at the end of the last two pregnancies. Three successful pregnancies were also reported in a patient with chronic renal disease whose primary disease was immunoglobulin A nephropathy. In that patient, the first conception occurred in the predialysis period, hemodialysis was started at the end of the first trimester of the second pregnancy, and she conceived for the third time in the second year of dialysis [10]. At the end of the each of the three pregnancies, the babies were discharged healthy.

The past three decades have seen substantial improvements in both fertility and successful pregnancy rates in women with chronic renal failure. Since the first case report of a successful pregnancy on dialysis in 1971 [11] and the case series reported in the 1980s that suggested high rates of preterm birth, low birth weight, and refractory hypertension, the overall prognosis for these patients has improved significantly [12]. Some evidence suggests that these improvements may be due to the recognition that a more intensive dialysis regimen may be beneficial. Uremia results in increased delivery of urea to the fetus and increases fetal solute load, which may lead to fetal diuresis and resultant polyhydramnios, thereby increasing risks of preterm birth and preterm rupture of membranes. To minimize the effects of uremia on pregnancy outcomes, dialysis regimens were adjusted with the goal of attaining maximum predialysis urea levels of 100 mg/dl or less. In subsequent case reports, authors have described improved perinatal outcome with intensive dialysis. Intensive dialysis regimens are believed to reduce the fetal uremic environment, minimize large fluid shifts and placental hypoperfusion, and allow greater dietary freedom with nutritional benefits [13]. In a systematic review [14], the relationship between dialysis schedule and pregnancy outcomes in pregnancies was analyzed in chronic dialysis in the new millennium. A total of 681 pregnancies were included in that review. The most frequently reported dialysis schedule was 3.5–4 h in five to six sessions per week. The authors of that review concluded that dialysis frequency and dialysis duration expressed as the number of hours per week were significantly correlated with two major outcomes: prematurity and delivery of a small for gestational age (SGA) baby (SGA defined as birth weight less than the tenth percentile according to international standards). The facts that my patient was young (with the first pregnancy at age 28 years and the second at 34) and her hypertension was not observed during follow-up, and that with her intensive hemodialysis regimen her blood urea level could mostly be kept below 100 mg/dl, might have contributed to the successful conclusions of her pregnancies.

## Conclusions

The most important factor affecting fetal and maternal prognosis is the degree of renal functional impairment at conception. A pregnancy with a successful outcome despite more than 10 years of dialysis, as in my patient, is very rarely reported in the literature. Her successful pregnancies were obtained by using an intensive hemodialysis regimen, appropriate anemia management, strict BP follow-up, and correct evaluation of her dry weight.

## Consent

Written informed consent was obtained from the patient for publication of this case report and any accompanying images. A copy of the written consent is available for review by the Editor-in-Chief of this journal.

## Abbreviations

BP: blood pressure
EDTA: European Dialysis and Transplant Association
ESRD: end-stage renal disease
IU: international units
SGA: small for gestational age
